# High Mortality from Blood Stream Infection in Addis Ababa, Ethiopia, Is Due to Antimicrobial Resistance

**DOI:** 10.1371/journal.pone.0144944

**Published:** 2015-12-15

**Authors:** Teshale Seboxa, Wondwossen Amogne, Workeabeba Abebe, Tewodros Tsegaye, Aklilu Azazh, Workagegnehu Hailu, Kebede Fufa, Nils Grude, Thor-Henrik Henriksen

**Affiliations:** 1 Department of Internal Medicine, School of Medicine, Addis Ababa University, Addis Ababa, Ethiopia; 2 Department of Pediatrics and Child Health, School of Medicine, Addis Ababa University, Addis Ababa, Ethiopia; 3 Department of Emergency and Critical Care Medicine, School of Medicine, Addis Ababa University, Addis Ababa, Ethiopia; 4 Department of Microbiology, Black Lion Hospital, Addis Ababa, Ethiopia; 5 Department of Microbiology, Vestfold Hospital Trust, Tönsberg, Norway; 6 Medical Department, Vestfold Hospital Trust, Tönsberg, Norway; McGill University Health Centre, CANADA

## Abstract

**Background:**

Managing blood stream infection in Africa is hampered by lack of bacteriological support needed for antimicrobial stewardship, and background data needed for empirical treatment. A combined pro- and retrospective approach was used to overcome thresholds in clinical research in Africa.

**Methods:**

Outcome and characteristics including age, HIV infection, pancytopenia and bacteriological results were studied in 292 adult patients with two or more SIRS criteria using univariate and confirming multivariate logistic regression models. Expected randomly distributed resistance covariation was compared with observed co-resistance among gram-negative enteric bacteria in 92 paediatric blood culture isolates that had been harvested in the same hospital during the same period of time.

**Results:**

Mortality was fivefold increased among patients with positive blood culture results [50.0% vs. 9.8%; OR 11.24 (4.38–25.88), p < 0.0001], and for this group of patients mortality was significantly associated with antimicrobial resistance [OR 23.28 (3.3–164.4), p = 0.002]. All 11 patients with *Enterobacteriaceae* resistant to 3^rd^. generation cephalosporins died. Eighty-nine patients had pancytopenia grade 3–4. Among patients with negative blood culture results, mortality was significantly associated with pancytopenia [OR 3.12 (1.32–7.39), p = 0.01]. HIV positivity was not associated with increased mortality. Antimicrobial resistance that concerned gram-negative enteric bacteria, regardless of species, was characterized by co-resistance between third generation cephalosporins, gentamicin, chloramphenicol, and co-trimoxazole.

**Conclusion:**

Mortality was strongly associated with growth of bacteria resistant to empirical treatment, and these patients were dead or dying when bacteriological reports arrived. Because of co-resistance, alternative efficient antibiotics would not have been available in Ethiopia for 8/11 *Enterobacteriaceae*-infected patients with isolates resistant to third generation cephalosporins. Strong and significant resistance covariation between 3^rd^. generation cephalosporins, chloramphenicol, gentamicin, and co-trimoxazole was identified. Pronounced pancytopenia was common and associated with increased mortality. HIV positive patients had no excess mortality.

## Introduction

During the past few decades, antimicrobial resistance has increased worldwide, and the perspectives are alarming [1, 2]. The nature, the magnitude and ways to cope with this problem are studied and described in the western world, while this base of knowledge is lacking in developing African countries [3–6]. We lack reports on mortality related to distribution of pathogens and their resistance patterns. Without such reports, guidelines for empiric treatment of severe bacterial diseases cannot be given. While updated studies on outcome in sepsis in Africa are almost non-existent, there are a few reports on bacterial culture results. The most alarming reports on antimicrobial resistance concern patients admitted to hospitals [7], while community-acquired infections may have lower profiles of resistance [8]. In Ethiopia, the resource situation has not allowed antimicrobial resistance to be prioritized as major public health concern despite the obvious needs [9–11]. There was a report of multi-resistant *Klebsiella spp*. in the paediatric department of Black Lion Hospital, Addis Ababa (BLH), in 1988 with resistance rates for chloramphenicol, gentamicin and co-trimoxazole of 96, 89 and 86%, respectively [10]. In the early nineties, the newly introduced third generation cephalosporins (3GC) cured 76% of severe bacterial infections not responding to standard antibacterial treatment at that time [12]. In 2006, the proportions of resistance were 86%, 83%, 3%, 86%, 9%, and 43% to ceftriaxone, chloramphenicol, ciprofloxacin, gentamicin, kanamycin and co-trimoxazole respectively, among 76 gram-negative blood culture (GNB) isolates from paediatric patients of the same hospital [11]. There are two recent reports about the sensitivity patterns of urinary tract pathogens in Ethiopia indicating alarming figures of resistance among GNB [13, 14]. According to these studies, the proportion of strains resistant towards ceftriaxone and ciprofloxacin has increased from 33 to 48% and 24 to 36%, respectively, from 2009 to 2014 [13]. Phenotypic beta-lactamase identification was performed in 17 consecutive *Enterobacteriaceae* isolates resistant to ceftazidime from BLH in 2013. All but one strain had extended spectrum beta-lactamase A (ESBL A). The remaining strain that did not carry ESBL A properties was resistant to meropenem (E-test method, authors’ unpublished observations). Later observations indicate that 2–3% of GNB isolates resistant towards 3^rd^. generation cephalosporins (3GC) are also meropenem resistant (authors’ unpublished observations). In a country where carbapenems have not yet been introduced, this is a remarkable observation. The clinical impression in Ethiopian hospitals is further that there is widespread resistance to 3GC, which is used empirically to treat blood stream infections. The daily concern is thus to seek alternatives for management of GNB infections. Much effort was therefore made to study covariation of resistance among GNB isolates.

Addis Ababa has 3,5 mill. inhabitants and is situated at 2300 meter above the sea level. Due to the high altitude, many diseases found in warm climates like malaria and visceral leishmaniasis are not spreading in Addis Ababa, but patients with these and other diseases are referred to our hospital from all parts of Ethiopia.

The aim was to study how mortality depended on bacterial pathogens, antimicrobial resistance, HIV coinfection and pancytopenia. In addition, covariation of resistance among GNB isolates was assessed. Datasets for the prospective (adult patients) and the retrospective (bacteriological reports, children´s specimens) are found at the end (S1 File)

## Materials and Methods

Data sampling was done in Black Lion Hospital (also called Tikur Anbessa Teaching Hospital), a large tertiary care hospital in Addis Ababa, Ethiopia, between August 2012 and October 2013. Adult patients were recruited when there was a clinical suspicion of septicaemia and 2 of the 3 following criteria were fulfilled: axillary temperature ≥ 38.5°C or ≤ 36.5°C, pulse ≥ 90 beats / minute and frequency of respiration ≥ 20 / minute. Patients who had received antibiotics within the last 72 hours were excluded. Clinical data of patients were recorded using a structured questionnaire. Laboratory results included HIV status (positive, negative or unknown). Haemoglobin (Hgb), White blood cell count (WBC), platelet count (Plt) and blood culture. Other investigations i.e. X-ray and clinical chemistry, were only done according to clinical need and are thus not reported. Blood cultures were collected with Bactec aerobic and anaerobic blood culture bottles (Becton, Dickinson and Company, 7 Loveton Circle Sparks, Maryland 21152, USA. One set (two bottles) only was taken from each patient. Bottles were kept at room temperature for up to 6 hours until transported to the International Clinical Laboratory, Addis Ababa, for incubation in a Bactec blood culture incubator at 35°C. Samples were recorded as negative when no growth was detected within five days’ incubation. Whenever there was clinical suspicion of endocarditis or slow growing microbes, incubation was prolonged to 10 days. Subculture was done on Oxoid´s blood and chocolate agar plates (Oxoid Limited, Wade Road, Basingstoke, Hampshire, RG248PW UK). Identification of species was started with gram stained smear. Fermentation profiles were used for gram negative aerobic rods while catalase test was done for gram positive cocci. For bacteria identified as *Staphylococcus spp*., Prolex test was applied (Pro-Lab, 3 Bassendale Road, Bromborough, Wirral, Merseyside, CH62 3QL, UK). Positive isolates were named *S*. *aureus* while negative isolates were labelled coagulase-negative *Staphylococcus* (CoNS). Further identification of *Staphylococci* was not done. Susceptibility testing of the non-fastidious isolates was done on Oxoid´s Müller-Hinton agar (CM0337). Oxoid susceptibility disks were used with Kirby-Bauer’s method throughout the study; zones of clearance were interpreted according to CLSI. Intermediate susceptibility was recorded as resistance. Susceptibility was measured for antimicrobials used in Ethiopia, including both ceftriaxone and cefotaxime but not ceftazidime and carbapenems.

### Statistical approaches

The needed statistical power and sample size was assessed with the intention to identify any difference in mortality ≥ 60% related to GNB sepsis with and without resistance to the empirically applied antibiotics—cefotaxime and ceftriaxone. Given α = 0,05 and 1-β = 0,80, the number of GNB isolates needed would be 20. If 50% of all isolates were GNB, we would need a total of 40 isolates. With expected yield of 15%, we would need 270 or more patients, to which we added a 10% safety margin. Sampling was terminated when 299 samples had been reached.

The odds ratio for variables´ association with outcome was first studied in a univariate model. For variables that had significant results, a multivariate model using logistic regression was applied to confirm significance. Because all patients with resistant GNB isolates died, OR and multiple regression could not be used for this group. Instead, relative risk was given.

Since beta lactamase-mediated resistance will not always be expressed phenotypically when tested for third generation cephalosporins, we tested for both ceftriaxone and cefotaxime while the reading with lowest sensitivity only was recorded [15]. For statistical calculations, intermediate resistance was counted as resistance.

Observed number of paediatric gram-negative isolates expressing resistance to 0–4 antibiotics (third generation cephalosporins, chloramphenicol, co-trimoxazole and gentamicin) was compared with expected randomly distributed co-resistance to assess the relative risk for covariation (N = 87).

### Classification of severe pancytopenia

We did not have the resources needed to make a definite diagnosis for patients with abnormal blood cell values. Instead, we analysed outcome for patients with grade 3–4 pancytopenia [16]. Patients found to have blood cell values clearly below what we would expect in septicaemia in Europe were recorded as having pancytopenia. The concerned patients were those with any one of the three cell lines being very low (Hgb < 7.0 g/100 ml, WBC <1.0 giga/L or Plt < 20 giga/L), when also the remaining two values were below a given limit, i.e. Hgb < 9,0 g/100 ml; WBC < 1.5 giga/L and Plt < 30 giga/L. We thus assessed the mortality risk attributable to strictly defined levels of pancytopenia.

### Retrospective study of paediatric isolates

In order to assess covariation of resistance among gram-negative isolates in Addis Ababa, we needed a larger number of isolates. This was made possible by reviewing bacteriological results of *Enterobacteriaceae* and non-fermenters in one year´s yield of positive blood culture results (October 2012 –September 2013) from children admitted to the Paediatric Department of BLH, Addis Ababa, Ethiopia. This was thus a retrospective study of blood samples in the same hospital at same time as the prospective study of adults. In contrast to the prospective clinical study on adult patients, these blood cultures were sampled and brought to the hospital’s bacteriological laboratory. Two to five mL of blood had been collected in bottles using conventional Brain-Heart-Infusion agar- based in-house method (Oxoid CM1135, Oxoid Limited, Wade Road, Basingstoke, Hampshire, RG248PW UK). They were incubated aerobically for 5 days without CO_2_ enrichment of atmosphere at 35°C. Subculture was done every other day. Subculture and susceptibility testing was performed as described above, with the exception that susceptibility to ceftazidime and amikacin was also tested for GNB in this cohort.

### Ethics statement

Ethical approval for the study was granted by the Institutional Review Board of Addis Ababa University Medical Faculty and from the Regional Committee for Medical and Health Research Ethics of Norway (REK sør-øst). Patients were included after receipt of written informed consent. For minors and unconscious patients consent was obtained from parents or guardians.

## Results

### Characteristics of survivors and non-survivors

Altogether 299 patients were included. Seven of these patients were later excluded because their blood culture samples had been lost on their way to the external laboratory. We have thus studied 292 adult patients. 164 (56%) were admitted to the medical ward while 126 (43%) were in emergency department of the medical admission unit. Two patients were recruited from the departments of surgery and gynaecology, respectively. Other candidates from these departments could not be included because they were all on antibiotics when they developed clinical signs of sepsis.

Overall mortality was 15.1% (44/292). Main characteristics of survivors and non-survivors are shown in Table 1.

**Table 1:** Characteristics of adult patients and the main results for survivors and non-survivors.

	All	Non-survivors N^°^ (%)	Survivors N^°^ (%)
All	292	44 (15)	248 (85)
Female	141	19 (43)	122 (49)
Median age Years [Range]	27 [13–98]	25 [13–70]	28 [13–98]
Median hospital days before blood culture	2	2	2
Blood culture growth	38	19 (50)	19 (50)
**HIV status**			
Positive	40	2 (5)	38 (95)
Negative	169	24 (14)	145 (86)
Unknown	83	18 (22)	65 (78)
**Haematological derangements**			
Pancytopenia	89	23 (26)	66 (74)
Hgb < 7,0	68	17 (25)	51 (75)
WBC <1,0	21	8 (38)	13 (62)
Platelets < 20	43	19 (44)	24 (56)

Altogether 38 blood cultures were positive, and the yield was thus 13,0%. The only three groups of bacteria identified were *Staphylococcus aureus* (7 patients), CoNS (11 patients) and GNB (20 patients). According to outcome of microscopy and catalase tests, none of the gram positive isolates were *Streptococci*. Patients with positive blood culture had a fivefold higher death rate than those without bacteraemia (50.0% vs. 9.8%). Details are given in Table 2.

**Table 2:** Odds Ratio for death attributable to bacteriological result, HIV co-infection, pancytopenia, age and AMR.

Variables	Dead	Alive	Univariate Analysis	Multivariate Analysis
			OR (95% CI)	OR (95% CI)
**All patients (N = 292)**				
Positive blood culture	19	19	9.16 (4.29–19.55)	11.24 (4.88–25.88)
HIV positive	2	38	5.26 (1.16–29.93)	3.77 (0.71–20.08)
Pancytopenia	23	66	3.02 (1.57–5.80)	3.30 (1.56–6.99)
Age			1.02 (0.99–1.05)	
**Patients with negative blood cultures (N = 254)**				
HIV positive	1	35	5.08 (0.62–41.79)	
Pancytopenia	14	63	3.35 (1.45–7.78)	3.12 (1.32–7.39)
Age			1.02 (0.99–1.05)	
**Patients with positive blood cultures (N = 38)**				
AMR	17	4	31.9 (5.1–199.5)	23.28 (3.3–164.4)
HIV positive	1	3	9.00 (0.66–122.8)	
Pancytopenia	9	3	4.80 (1.04–22.10)	3.65 (0.50–26.59)
Age			1.03 (0.99–1.07)	
**Patients with growth of gramnegative enteric bacteria (N = 20)**				
AMR	11	0	Not done	
HIV positive	1	3	12.00 (0.5–280.1)	
Pancytopenia	8	3	3.33 (0.52–21.58)	
Age			1.03 (0.98–1.09)	

AMR: Resistance to 3^rd^. generation Cephalosporins.

One patient with *S*. *aureus* septicaemia died. The clinical picture of this 25 years old patient was consistent with advanced endocarditis with cerebral embolus and the isolate was fully sensitive towards all antibiotics that had been tested. Except for this patient with probable complicated endocarditis, all patients with *S*. *aureus* related sepsis recovered and were discharged alive. Five of the *S*. *aureus* isolates were fully susceptible to ceftriaxone, cefotaxime and cephalothin. One isolate expressed susceptibility to cephalothin and cefotaxime and intermediate susceptibility to ceftriaxone, while the seventh isolate was resistant towards ceftriaxone. One isolate expressed intermediate susceptibility to vancomycin (Table 3). The significance of this observation is discussed.

**Table 3:** Resistance profiles and outcomes for patients with *S*. *aureus* sepsis

Organism	CRO	CTX	Outcome	AmC	CIP	GM	SXT	CC	E	Va
*S*. *aureus*	S	S	Discharged	S	S	S	I	I	I	I
*S*. *aureus*	S	S	Discharged	S	S	S	S	S	S	S
*S*. *aureus*	S	S	Discharged	S	S	S	I	S	S	S
*S*. *aureus*	S	S	Discharged	S	S	S	R	S	S	S
*S*. *aureus*	S	S	Died	S	S	S	S	S	S	S
*S*. *aureus*	I	S	Discharged	S	S	I	R	S	S	S
*S*. *aureus*	R	I	Discharged	S	S	S	R	R	S	S

CRO, Ceftriaxone; CTX, Cefotaxime; AmC, Amoxicillin + Clavulanic acid; CIP, Ciprofloxacin; GM, Gentamycin; SXT, Sulfamethoxazole + Trimethoprim (Co-trimoxazole); CC, Clindamycin; E, Erythromycin; Va, Vancomycin

For patients with GNB sepsis, mortality was strongly associated with resistance to 3GC (Table 4). In fact, all patients died whose isolate had reduced sensitivity to 3GC. Among patients with 3GC sensitive isolates, one patient died. Because there were zero survivors among patients with 3GC resistant isolates, univariate and multivariate logistic regression analysis with odds ratio could not be done. Instead, relative risk was calculated [11/11 vs. 1/9; RR 9.00 (1.42–57.12, p = 0.0198)]. The single patient who died from GNB sepsis with a fully sensitive isolate had been in hospital for a condition that appeared to be acute leukaemia. This patient was included in the study when sepsis developed 7 days after admission.

**Table 4:** Antimicrobial resistance, pancytopenia and outcome for patients with sepsis caused by gram-negative rod-shaped bacteria.

Organism	CRO	CTX	Pancytopenia	Outcome	AmC	CIP	GM	SXT
*E*. *coli*	R	R	Yes	Died	R	R	R	R
*E*. *coli*	R	R	Yes	Died	R	R	R	R
*E*. *coli*	R	R	Yes	Died	R	R	R	R
*E*. *coli*	R	R	No	Died	R	R	R	R
*E*. *coli*	R	R	Yes	Died	R	I	R	R
*E*. *coli*	R	R	Yes	Died	R	I	R	R
*E*. *coli*	R	R	Yes	Died	I	R	S	R
*E*. *coli*	R	R	No	Died	I		R	R
*Klebsiella spp*.	R	R	Yes	Died	I	R	R	R
*E*. *coli*	I	I	Yes	Died	S	I	S	
*E*. *coli*	S	I	No	Died	S	I	S	R
*Salmonella spp*.	S	S	Yes	Discharged	S	S	S	S
*Salmonella spp*.	S	S	No	Discharged	S	S	S	S
*C*. *braakii*	S	S	No	Died	S	S	S	
*E*. *coli*	S	S	Yes	Discharged	R	R	R	S
*E*. *coli*	S	S	No	Discharged	S	R	S	R
*E*. *coli*	S	S	No	Discharged	I	S	S	S
*E*. *coli*	S	S	No	Discharged	S	S	S	R
*E*. *coli*	S	S	No	Discharged	S	S	S	S
*E*. *coli*	S	S	Yes	Discharged	R	S		R

CRO, Ceftriaxone; CTX, Cefotaxime; AmC, Amoxicillin + Clavulanic acid; CIP, Ciprofloxacin; GM, Gentamycin; SXT, Sulfamethoxazole + Trimethoprim (Co-trimoxazole); R, Resistance; I, Intermediate susceptibility; S, Susceptibility

Mortality among patients with coagulase negative Staphylococci (CoNS) was 6/11 = 54.5%, which was significantly higher than mortality recorded for patients with negative blood culture results (RR = 5.54; CI 2.88–10.67).

### Co-variation of resistance

The tendency was that GNB isolates expressed reduced susceptibility to non-beta-lactam antibiotics when isolates were also resistant to cefotaxime or ceftriaxone. In fact, efficient alternatives would not have been available in Ethiopia for 8/11 patients who died with Enterobacteriaceae-infected isolates that had low susceptibility or resistance to 3GC (Table 4).

Covariation of resistance was studied more extensively in the paediatric cohort, and these results are given in Tables 5 and 6. Susceptibility results for one antimicrobial agent could not be retrieved for five of the isolates, and these are excluded from Table 6. Test results from all 92 isolates are included in Table 5, which gives extent of covariation as percent. While Table 6 expresses the extent of group covariation, Table 5 gives the rate of agreement between 3GC and one other agent at the time. As can be seen in Table 5, >70% of isolates expressed resistance covariation between 3GC, chloramphenicol, co-trimoxazole, tetracycline and gentamicin. This property was not confined to one single strain, but was shared by all groups of gram-negative rods that were tested. Ciprofloxacin and amikacin were the exceptions. Ninety percent of isolates expressed susceptibility towards amikacin.

**Table 5:** Covariation of resistance between ceftazidime and 6 other antibiotics. Covariation is given as agreement on resistance and susceptibility, respectively, between ceftazidime and one other agent at the time. (100% = full agreement). Four different groups of gram-negative bacteria. Paediatric patients.

	Non-Klebsiella Enterobacteriaceae		*Klebsiella spp*.		*Pseudomonas spp*.		*Acinetobacter spp*.		All	
**Ceftazidime** (Number of isolates)	R = 18	S = 11	R = 29	S = 5	R = 6	S = 5	R = 11	S = 7	R = 64	S = 28
**Gentamicin**	83%	100%	93%	20%	83%	100%	64%	100%	84%	85%
**Chloramphenicol**	44%	100%	76%	75%	83%	20%	91%	72%	71%	74%
**Tetracyclin**	89%	36%	88%	60%	67%	80%	64%	67%	82%	56%
**Co-trimoxazole**	94%	73%	83%	60%	50%	80%	80%	86%	82%	75%
**Ciprofloxacin**	67%	100%	31%	60%	0%	100%	45%	100%	39%	83%
**Amikacin**	8%	100%	4%	60%	32%	50%	10%	100%	10%	81%

R, Intermediate susceptibility or resistance; S, Full susceptibility.

**Table 6:** Covariation of resistance to third generation cephalosporins, chloramphenicol, co-trimoxazole and gentamicin among gram-negative bacteria, paediatric cohort. Observed number of isolates expressing resistance to 0–4 antibiotics compared with expected randomly distributed co-resistance (N = 87).

Frequencies	Expected	Observed (CI)	Relative risk
0	1.8	15 (8.7–23.3)	8.3
1	11.9	11 (5.6–18.7)	0.9
2	29.4	8 (3.5–15.1)	0.3
3	31.5	24 (16.1–33.2)	0.8
4	12.5	29 (20.5–38.5)	2.3

Table 6 shows the mathematical expression of covariation between four different groups of antibiotics. The observed numbers of isolates expressing resistance to 0 and 4 antibiotics were significantly above expected numbers.

### Significance of HIV infection and pancytopenia

Eighty-nine patients (30,5%) had pancytopenia as defined here. Mortality was significantly increased for pancytopenic patients, and this significance remained in the multivariate analysis among patients with negative blood culture results. Pancytopenia was also associated with significantly increased mortality among patients with positive blood cultures in the univariate analysis, but this association was not confirmed in the multivariate analysis. None of the patients with *S*. *aureus* infection, one patient with CoNS and 11/20 patients (55%) with GNB septicaemia had pancytopenia.

In all but four of the HIV positive patients, i.e. thirty-six cases, patients were thought to have AIDS on clinical grounds. Only two of these patients died, however, and HIV positives had significantly lower mortality than others (HIV negatives and patients who had not been tested for HIV) in the univariate analysis. The significance did not remain in the multivariate analysis, however (Table 2).

## Discussion

### Mortality associated with positive blood culture results

The mortality risk was increased fivefold in those with a positive blood culture. The most notable observation was that reduced sensitivity towards 3GC, the empirically used antibiotics, could not be overcome in sepsis with GNB. All 11 patients with resistant GNB died, which alone represented 25% of the reported deaths in this study. Growth of CoNS was also associated with increased mortality.

Based on bacteriological observations, patients with positive blood cultures consisted of 3 different groups with different clinical pictures and outcomes: seven patients with *S*. *aureus*, eleven with CoNS and twenty with GNB bacteraemia.

The isolate of the one patient who died with *S*. *aureus* sepsis was fully susceptible to all tested antibiotics. It is likely that prognosis was poor because of advanced disease when the patient arrived for admission. The other 6 patients with *S*. *aureus* sepsis improved on treatment, and the in vitro susceptibility cards revealed susceptibility towards three or more non-betalactam antibiotics for all isolates including the two mentioned in Table 3 that had reduced sensitivity to cephalosporins.

In the group of patients with GNB sepsis, mortality was strongly associated with susceptibility patterns. The absence of early laboratory reports in patients with GNB sepsis made antimicrobial stewardship impossible. However, even if the resistance patterns had been known earlier, it could still have been very difficult to save a number of patients. As shown in Table 4, resistance to 3GC was commonly accompanied by resistance to alternative antibiotics as well. For most of these patients, efficient antimicrobials would not be found on the Ethiopian market.

The third group of patients with positive blood cultures were patients with CoNS isolates. CoNS-related mortality usually concerns endocarditis and infections related to foreign bodies e.g. prostheses [17]. None of our patients had prostheses or other foreign bodies. Endocarditis was only suspected in one patient with growth of CoNS in blood culture. CoNS commonly represents skin micro flora contamination when found in blood cultures in high-income countries. Contamination is most likely to occur when phlebotomy is difficult, as in dehydrated and heavily diseased cases. In countries that have reduced health resources, disease severity is a stronger predictor of poor outcome than elsewhere [17]. We have no proof for this in our study, however. One of the 89 pancytopenic patents had CoNS in his blood culture. While serum creatinine was normal for three of the patients who died, it had not been checked for the remaining three patients who died.

In this limited study *S*. *aureus* resistance was not a challenge, while inability to manage infections caused by resistant GNB was a huge problem. Blood stream infection (BSI) with virulent *Enterobacteriaceae* is a potentially deadly condition, and survival depends on capacity of caretaking institution to manage clinical challenges including organ failure and antimicrobial resistance [18–22]. Standards for management of these patients are developed in well-equipped western hospitals with well-manned clinical units, good laboratories and well-developed routines for communication and interaction between professional groups. When availability of resources is low, capacity to reach these demands is limited. In a situation like ours where antimicrobial stewardship could not be achieved, we would expect survival in BSI to depend on two things: treatment that is started before severe organ failure has developed, and pathogens that are susceptible to the antibiotic that is given empirically [23]. It is a tragedy, of course, if empirical treatment commonly fails in settings where resistance cannot be identified in time. Hospitals that lack laboratories for bacteriology are in urgent need of sufficiently equipped and manned laboratories. According to the present study, amikacin should be imported and become a part of empirical treatment of BSI in Ethiopia. However, heavy decisions about treatment guidelines for BSI should rest upon a much wider base of information: we need more studies to find the most appropriate antibiotics for empirical use.

### HIV infection

HIV infection and pancytopenia were found in a large number of patients (123 = 42%) While pancytopenia contributed to the background mortality, HIV infection did not. When knowing that a septic patient was HIV positive, the common assumption was that this patient had also AIDS. With reference to the low mortality rate in this cohort, this assumption was probably commonly wrong. We suggest that CD4 cell counts are made for all HIV positives in future sepsis studies in Africa. In Ethiopia, efficient HIV management is now available. Most patients with known HIV infection can thus be protected against disease progression [24]. When there is awareness about HIV infection, this coinfection may not be a major mortality risk. Immune deficiency may be more common among those who have not been tested for HIV infection [24].

### Pancytopenia

Medicine commonly needs to be redefined to become manageable in resource-poor settings. The approach used in this study adopted the strategy of simplifying the following elements to make research manageable:

1. At this time point, the focus is on major causes of death—i.e. those that are exposed even with moderate statistical power [25].
2. The most demanding part was to evaluate and follow patients. A large patient cohort, which would have been beyond our capacity, would be needed to study resistance covariation. This was overcome by exchanging the additional need for patients with a retrospective review of contemporary laboratory results in our hospital.
3. Comorbidity scores were not assessed because they were found to be non-applicable in a resource-poor setting.

Comorbidity influences risks for infection and death [26, 27]. However, even in newer versions, comorbidity index systems are not adopted to clinical risks in Africa, and clinical diagnoses in Africa are commonly not sufficiently structured for calculation of comorbidity scores [28]. Many of our patients had abnormal haematological values, and 89 patients had severe pancytopenia. In Europe, severe pancytopenia is commonly seen in haematological malignancies and only occasionally observed as caused by bacterial infection [29, 30]. This may be different in in Africa. To what extent pancytopenia was caused by sepsis or a haematological comorbidity could not be answered, but that may not be very important: While studying impact of antimicrobial stewardship on sepsis, it is important to measure variables that contribute to mortality also when the bacterial infection is treated correctly. Pancytopenia can be used for this purpose in Africa.

### Laboratory methodology and epidemiology of antimicrobial resistance

The observed intermediate susceptibility to vancomycin in one *S*. *aureus* isolate in this study was unexpected. Vancomycin susceptibility testing is challenging, and the isolate was unfortunately not retested. There have been some reports on reduced susceptibility to vancomycin among *Staphylococc spp*. in Asian developing countries. *Staphylococcus* isolates showing reduced susceptibility to vancomycin should therefore be retested with improved methodology [31].

Sensitivity testing of GNB with the methods used here is debatable as resistance to 3GC may not be expressed in vitro. This concerns *Enterobacter spp*. of course, but also other GNB groups may have a lower sensitivity than the inhibition zone would say. When it comes to 3GC readings in GNB isolates, we therefore prefer to record readings of intermediate sensitivity as resistant. This was also done in the present study.

During the past ten years, there has been one previous report on outcome of BSI among adult patients in Africa [32]. That study focuses on the impact of HIV management. There are a few studies on BSI in childhood, most of them concerning neonates [33–36]. The common finding is that antimicrobial resistance is an important killing factor among children in all age groups, and that resistant GNB are the worst (5–6).

The risks for selection and spread of resistance are high whenever 3GC is used. Once resistance has started to spread, continuous exposure to the same antibiotic may worsen the situation. The observation that covariation of resistance is not confined to one species, but shared by all groups of GNB tested is worth noticing (Table 5). If our observation of resistance covariation is confirmed, continuous use of any one of these five compounds will ease propagation of resistance to all of them [30]. It is important that antibiotics are used with restriction and care, and that chloramphenicol, 3GC, co-trimoxazole, tetracycline and gentamicin are all avoided as much as possible. Resistance towards ciprofloxacin is already an increasing problem. Nevertheless, the impression is that ciprofloxacin and amikacin are not part of this covariation (Table 5). The same is probably also true for carbapenems that were not tested here. It is sad that resistance to carbapenems seems to be spreading even before carbapenems have been used in the country (authors’ unpublished observation). Introduction of carbapenems may now trigger further accumulation of carbapenem resistance in Ethiopia [37]. In the present situation, amikacin is probably the best alternative for empiric coverage against GNB. More than 90% of Gram negative isolates are susceptible towards amikacin, and its barrier against development of resistance is probably higher than for any alternative. Previous experience shows that caution is highly needed even with this antibiotic, as amikacin resistance may also be aggregated [38]. When used empirically for BSI, amikacin should be combined with another compound that needs to be discussed upon.

There is growing evidence for global spread of antimicrobial resistance [39]. Even if the burden of antimicrobial resistance in western countries like Norway is much smaller than in Ethiopia, rates are increasing exponentially, and may become a major problem in western countries as well [40].The identification of meropenem-resistant *K pneumonia* in Addis Ababa before carbapenems were used inside the country can be due to the health care tourism of recent years. The Ethiopian middle class, now counted in millions, attends hospitals in Thailand and other destinations overseas for medical treatment including surgery. Thus, Ethiopia is probably a receiver of this severe health problem. At the same time, this and other studies from Ethiopia point at the current accumulation of antimicrobial resistance inside Ethiopia itself. The world is woven together in a network of nations that are receivers, producers, and exporters of antimicrobial resistance: concentrated resistance in one corner of the world will become a global problem [41, 42]. This and other papers contribute to the understanding that mapping, managing and halting resistance is mostly needed and most difficult where resources are scarce. Resource poverty in developing countries has thus become our common enemy [2, 43].

## Supporting Information

S1 File Dataset used for the article (XLSX)

## References

[1] Antimicrobial Resistance: Global Report on Surveillance. Geneva: World Health Organization; 2014 256 s. p.

[2] WHO: Antimicrobial resistance a global threat. Drug Ther Bull. 2014;52(7):77.

[3] MacVane SH, Tuttle LO, Nicolau DP. Impact of extended-spectrum beta-lactamase-producing organisms on clinical and economic outcomes in patients with urinary tract infection. Journal of hospital medicine: an official publication of the Society of Hospital Medicine. 2014;9(4):232–8. Epub 2014/01/28. 10.1002/jhm.2157 . 24464783

[4] Leistner R, Gurntke S, Sakellariou C, Denkel LA, Bloch A, Gastmeier P, et al Bloodstream infection due to extended-spectrum beta-lactamase (ESBL)-positive K. pneumoniae and E. coli: an analysis of the disease burden in a large cohort. Infection. 2014;42(6):991–7. Epub 2014/08/08. 10.1007/s15010-014-0670-9 . 25100555

[5] Leistner R, Sakellariou C, Gurntke S, Kola A, Steinmetz I, Kohler C, et al Mortality and molecular epidemiology associated with extended-spectrum beta-lactamase production in Escherichia coli from bloodstream infection. Infect Drug Resist. 2014;7:57–62. Epub 2014/03/22. 10.2147/idr.s56984 ; PubMed Central PMCID: PMCPmc3958498. 24648746 PMC3958498

[6] Willmann M, Kuebart I, Marschal M, Schroppel K, Vogel W, Flesch I, et al Effect of metallo-beta-lactamase production and multidrug resistance on clinical outcomes in patients with Pseudomonas aeruginosa bloodstream infection: a retrospective cohort study. BMC Infect Dis. 2013;13:515 Epub 2013/11/02. 10.1186/1471-2334-13-515 ; PubMed Central PMCID: PMCPmc3818928. 24176052 PMC3818928

[7] Vlieghe E, Phoba MF, Tamfun JJM, Jacobs J. Antibiotic resistance among bacterial pathogens in Central Africa: a review of the published literature between 1955 and 2008. Int J Antimicrob Agents. 2009;34(4):295–303. 10.1016/j.ijantimicag.2009.04.015 . 19540725

[8] Ashley EA, Lubell Y, White NJ, Turner P. Antimicrobial susceptibility of bacterial isolates from community acquired infections in Sub-Saharan Africa and Asian low and middle income countries. Trop Med Int Health. 2011;16(9):1167–79. 10.1111/j.1365-3156.2011.02822.x 21707879 PMC3469739

[9] Lindtjorn B, Setegn D, Niemi M. Sensitivity patterns of bacteria isolated from patients at Sidamo Regional Hospital. Ethiop Med J. 1989;27(1):27–31. . 2920709

[10] Moss W. An outbreak of gentamicin-resistant Klebsiella bacteraemia at a children's hospital. Ethiop Med J. 1992;30(4):197–205. . 1459119

[11] Shitaye D, Asrat D, Woldeamanuel Y, Worku B. Risk factors and etiology of neonatal sepsis in Tikur Anbessa University Hospital, Ethiopia. Ethiop Med J. 2010;48(1):11–21. . 20607993

[12] Oli K, Haimanot R, Gedebou M, Tsega E. Ceftriazone in the treatment of septicaemia due to multipledrug resistant bacteria. Cent Afr J Med. 1990;36(3):68–71. 2225021

[13] Kindie AD DM, Markussen C, Johansen OH, Grude N. High and Increasing Cephalosporin and Fluoroquinolone Resistance in Uropathogenic E. Coli in Addis Ababa, Ethiopia. 31st World Congress of Biomedical Laboratory Science. 2014. 31st World Congress of Biomedical Laboratory Science.

[14] Alemu A, Moges F, Shiferaw Y, Tafess K, Kassu A, Anagaw B, et al Bacterial profile and drug susceptibility pattern of urinary tract infection in pregnant women at University of Gondar Teaching Hospital, Northwest Ethiopia. BMC Res Notes. 2012;5:197 10.1186/1756-0500-5-197 22534117 PMC3473254

[15] Willems E, Verhaegen J, Magerman K, Nys S, Cartuyvels R. Towards a phenotypic screening strategy for emerging beta-lactamases in Gram-negative bacilli. Int J Antimicrob Agents. 2013;41(2):99–109. 10.1016/j.ijantimicag.2012.07.006 23280443

[16] Common Terminology Criteria for Adverse Events (CTCAE) Version 4.0. (v.403: June 14, 2010). May 28, 2009 ed: US Department of Health and Human Services.; 2010.

[17] Archer GLCMW. *Staphylococcus epidermidis* and Other Coagulase-Negative Staphylococci In: Mandell ID, Bennett G.L., G R., editor. PRINCIPLES AND PRACTICE OF INFECTIOUS DISEASES. II. Sixth ed Philadelphia, PA, USA: Elsevier Churchill Livingstone; 2005 p. 2352–4.

[18] Law BJ, Rettig PJ, Marks MI. Antibiotic therapy of fulminant E. coli K1 sepsis in infant rabbits. Pediatr Res. 1984;18(4):314–7. Epub 1984/04/01. . 6371694 10.1203/00006450-198404000-00002

[19] Kumar A, Haery C, Paladugu B, Kumar A, Symeoneides S, Taiberg L, et al The duration of hypotension before the initiation of antibiotic treatment is a critical determinant of survival in a murine model of Escherichia coli septic shock: association with serum lactate and inflammatory cytokine levels. J Infect Dis. 2006;193(2):251–8. Epub 2005/12/20. 10.1086/498909 . 16362889

[20] Osler W. Septicæemia and pyæmia In: Osler W, editor. The principles and practice of Medicine. New York, USA: D. Appleton and company,; 1892 p. p 114–5.

[21] Ani C, Farshidpanah S, Bellinghausen Stewart A, Nguyen HB. Variations in organism-specific severe sepsis mortality in the United States: 1999–2008. Crit Care Med. 2015;43(1):65–77. Epub 2014/09/18. 10.1097/ccm.0000000000000555 . 25230374

[22] Laupland KB, Gregson DB, Zygun DA, Doig CJ, Mortis G, Church DL. Severe bloodstream infections: a population-based assessment. Crit Care Med. 2004;32(4):992–7. Epub 2004/04/09. . 15071391 10.1097/01.ccm.0000119424.31648.1e

[23] Cheng WL, Hsueh PR, Lee CC, Li CW, Li MJ, Chang CM, et al Bacteremic pneumonia caused by extended-spectrum beta-lactamase-producing Escherichia coli and Klebsiella pneumoniae: Appropriateness of empirical treatment matters. Journal of microbiology, immunology, and infection = Wei mian yu gan ran za zhi. 2014 Epub 2014/07/30. 10.1016/j.jmii.2014.05.003 . 25070279

[24] Biadgilign S, Reda A, Digaffe T. Predictors of mortality among HIV infected patients taking antiretroviral treatment in Ethiopia: A retrospective cohort study. AIDS Res Ther. 2012;9 10.1186/1742-6405-9-15 PMC3403909 22606951

[25] Rothman KJ. Objectives of epidemiologic study design In: Rothman KJ, editor. Modern epidemiology. 1. Boston/Toronto: Little, Brown and Company; 1986 p. 77–97.

[26] Charlson ME, Sax FL, MacKenzie CR, Braham RL, Fields SD, Douglas RG Jr. Morbidity during hospitalization: can we predict it? J Chronic Dis. 1987;40(7):705–12. Epub 1987/01/01. . 3110198 10.1016/0021-9681(87)90107-x

[27] Charlson ME, Pompei P, Ales KL, MacKenzie CR. A new method of classifying prognostic comorbidity in longitudinal studies: development and validation. J Chronic Dis. 1987;40(5):373–83. Epub 1987/01/01. . 3558716 10.1016/0021-9681(87)90171-8

[28] McGregor J, Kim P, Perencevich E, Bradham D, Furuno J, Kaye K, et al Utility of the Chronic Disease Score and Charlson Comorbidity Index as comorbidity measures for use in epidemiologic studies of antibiotic-resistant organisms. Am J Epidemiol. 2005;161(5):483–93. 10.1093/aje/kwi068 15718484

[29] Ito S, Takada N, Ozasa A, Hanada M, Sugiyama M, Suzuki K, et al Secondary hemophagocytic syndrome in a patient with methicillin-sensitive Staphylococcus aureus bacteremia due to severe decubitus ulcer. Intern Med. 2006;45(5):303–7. 10.2169/internalmedicine.45.1535 16595999

[30] Steinbigler P, Wichert V, Manz C, Hofling B. Pancytopenia and septic temperatures in a 29-year-old female patient one year after visiting the Mediterranean Sea. Internist. 1995;36(6):608–10. 7665330

[31] Chen C, J., Huang Y, C. New epidemiology of Staphylococcus aureus infection in Asia. Clinical Microbiology and Infection. 2014;20(7):605–23. 10.1111/1469-0691.12705 24888414

[32] Feasey NA, Houston A, Mukaka M, Komrower D, Mwalukomo T, Tenthani L, et al A reduction in adult blood stream infection and case fatality at a large African hospital following antiretroviral therapy roll-out. PLoS One. 2014;9(3):e92226 Epub 2014/03/20. 10.1371/journal.pone.0092226 ; PubMed Central PMCID: PMCPmc3958486. 24643091 PMC3958486

[33] Blomberg B, Jureen R, Manji KP, Tamim BS, Mwakagile DS, Urassa WK, et al High rate of fatal cases of pediatric septicemia caused by gram-negative bacteria with extended-spectrum beta-lactamases in Dar es Salaam, Tanzania. J Clin Microbiol. 2005;43(2):745–9. 10.1128/JCM.43.2.745-749.2005 15695674 PMC548071

[34] Blomberg B, Manji KP, Urassa WK, Tamim BS, Mwakagile DSM, Jureen R, et al Antimicrobial resistance predicts death in Tanzanian children with bloodstream infections: A prospective cohort study. BMC Infectious Diseases. 2007;7(46). 10.1186/1471-2334-7-43 PMC1891109 17519011

[35] Velaphi S, Wadula J, Nakwa F. Mortality rate in neonates infected with extended-spectrum beta lactamase-producing Klebsiella species and selective empirical use of meropenem. Ann Trop Paediatr. 2009;29(2):101–10. Epub 2009/05/23. 10.1179/146532809x440716 . 19460263

[36] Schrag SJ, Cutland CL, Zell ER, Kuwanda L, Buchmann EJ, Velaphi SC, et al Risk factors for neonatal sepsis and perinatal death among infants enrolled in the prevention of perinatal sepsis trial, Soweto, South Africa. Pediatric Infectious Disease Journal. 2012;31(8):821–6. 10.1097/INF.0b013e31825c4b5a 22565291

[37] Cuervo SI, Sanchez R, Gomez-Rincon JC, Almenares C, Osorio JP, Vargas MJ. [Behavior of carbapenemase-producing Klebsiella pneumoniae cases in cancer patients at a third level hospital in Bogota, D.C]. Biomedica (Bogota). 2014;34 Suppl 1:170–80. Epub 2014/06/27. 10.1590/s0120-41572014000500020 . 24968049

[38] Friedland IR, Funk E, Khoosal M, Klugman KP. Increased resistance to amikacin in a neonatal unit following intensive amikacin usage. Antimicrob Agents Chemother. 1992;36(8):1596–600. 1416839 10.1128/aac.36.8.1596 PMC192005

[39] Shallcross LJ, Davies SC. The World Health Assembly resolution on antimicrobial resistance. J Antimicrob Chemother. 2014 10.1093/jac/dku346 . 25204342

[40] NORM-VET. NORM/NORM-VET: consumption of antimicrobial agents and occurrence of antimicrobial resistance in Norway. [Tromsø] [Oslo]: [NORM, Department of Microbiology [NORM-VET.

[41] Kuenzli E, Jaeger VK, Frei R, Neumayr A, DeCrom S, Haller S, et al High colonization rates of extended-spectrum beta-lactamase (ESBL)-producing Escherichia coli in Swiss Travellers to South Asia- a prospective observational multicentre cohort study looking at epidemiology, microbiology and risk factors. BMC Infect Dis. 2014;14:528 Epub 2014/10/02. 10.1186/1471-2334-14-528 . 25270732 PMC4262238

[42] Tham J, Odenholt I, Walder M, Andersson L, Melander E. Risk factors for infections with extended-spectrum beta-lactamase-producing Escherichia coli in a county of Southern Sweden. Infect Drug Resist. 2013;6:93–7. 10.2147/IDR.S46290 24082789 PMC3785393

[43] Laxminarayan R, Duse A, Wattal C, Zaidi AK, Wertheim HF, Sumpradit N, et al Antibiotic resistance-the need for global solutions. Lancet Infect Dis. 2013;13(12):1057–98. 10.1016/S1473-3099(13)70318-9 24252483

